# PEOPLE LIVING WITH DEMENTIA GUIDED THE WAY: ADAPTIVE STAKEHOLDER ENGAGEMENT TO DESIGN A PRAGMATIC CLINICAL TRIAL

**DOI:** 10.1093/geroni/igac059.1816

**Published:** 2022-12-20

**Authors:** Michael Lepore

**Affiliations:** University of Maryland School of Nursing, Baltimore, Maryland, United States

## Abstract

Research on non-pharmacologic interventions for people living with dementia (PLWD) has shown many benefits, but healthcare systems do not offer these interventions widely. To strengthen understanding of the real-world effectiveness of evidence-based non-pharmacologic interventions for PLWD requires a pragmatic approach to trialing interventions in the contexts of healthcare systems where they are standardly delivered. Designing pragmatic trials with the engagement of primary stakeholders can support trial feasibility and meaningful outcome measurement, but little evidence is available on approaches for engaging PLWD in designing pragmatic trials. This longitudinal case study introduces adaptive stakeholder engagement, a three-phase approach to engaging PLWD in planning a pragmatic trial. First, a multistakeholder workshop including PLWD was held where participants prioritized topics for research, including research on the impact of increasing social activity. Second, an evidence-based non-pharmacological intervention that addresses this priority area was piloted with PLWD and qualitative feedback was collected from PLWD over the course of intervention piloting to inform the trial design. Finally, PLWD were engaged in facilitated monthly meetings to provide input on the pragmatic trial design. In addition to informing intervention selection, input collected from PLWD informed the pragmatic trial in several ways, including approaches to PLWD recruitment, disclosure of research to PLWD, and intervention orientation processes. The adaptive stakeholder engagement model involved PLWD in different roles, as workshop participants, intervention participants, and trial advisors. This model enabled PLWD to be engaged in priority setting, infrastructure development, and trial protocol design, and may be useful for researchers planning future pragmatic trials.

